# Implementing TB-Stigma reduction interventions in High Burden Countries: a perspective on a conceptual framework

**DOI:** 10.3389/ftubr.2025.1721361

**Published:** 2025-11-28

**Authors:** Bushra Bibi, Mark Goodall, Injety Ranjit, Stephanie Jones, Colette Miller

**Affiliations:** 1 Office of the Research Innovation & Commercialization, Khyber Medical University, Peshawar, Pakistan; 2 Primary Care and Mental Health, Institute of Population Health, University of Liverpool, Liverpool, United Kingdom; 3 School of Nursing and Midwifery and the Applied Health Research Hub (AHRh), University of Central Lancashire, Preston, United Kingdom; 4 Ulverscroft Eye Unit, Institute of Precision Health, University of Leicester, Leicester, United Kingdom; 5 Stroke Research Team, School of Nursing & Midwifery, University of Central Lancashire, Preston, United Kingdom

**Keywords:** tuberculosis, stigma, intervention, conceptual framework, implementation

## Abstract

Tuberculosis (TB) is a preventable, curable disease but still impacts people in high-burden countries, who face challenges, including stigma, in accessing and engaging with healthcare services. The Nuttall's review examined the quality of existing TB-stigma intervention studies and developed a conceptual framework of pathways to stigma reduction. We critically appraised the methods used and expanded upon these findings in the context of the WHO End TB Strategy. The included studies showed significant heterogeneity in design, aims, populations, type of TB-stigma targeted and took place across diverse countries. Only three of 11 studies were rated as high quality. This systematic review synthesized existing interventions and outcomes into a conceptual framework outlining pathways to reduce TB-stigma. The conceptual framework highlights the need for educational, emotional, and psychosocial support for TB patients, Health Care Workers, and Communities, and provides a useful guide of pathways needed in TB-stigma reduction interventions. However, to be effective, stigma reduction interventions must be part of a well-organized, and committed multi-sectoral collaboratives, which extend beyond national and global TB programs, including mental health services, social support systems, and public health programs.

## Introduction

Each year, approximately 10 million people contract tuberculosis (TB), a preventable and curable disease (1). The World Health Organization (WHO) End TB Strategy's goal is to reduce the global TB epidemic by 2035 (2). Half of all High Burden Countries (HBC) for TB, as defined by WHO, are listed as low- and middle-income countries (LMICs) (1). People with TB still face challenges in accessing, and engaging with healthcare services (3).

Stigma is described by the WHO as a “hidden” burden of disease (4) and a major barrier to ending TB globally (5). People with TB often face different types of stigmas as defined in Box 1. These stem from cultural fears and misconceptions, which significantly hinder testing, contact tracing, treatment linkage, and medication adherence (6, 7). However, the process and impact evaluation of TB-stigma interventions are limited. A 2017 review focusing on stigma-reduction interventions highlighted the complexity of measuring and addressing stigma (9). Recently, Nuttall et al. (10) examined the quality of existing TB-stigma intervention studies and created a novel conceptual framework of pathways to TB-stigma reduction (10). In this perspective we critically analyse Nuttall et al. (10) and apply their findings in the context and the aims of the WHO End TB Strategy. We ask the question can this conceptual framework help HBC for TB to operationalise effective TB-stigma reduction programs. The Nuttall et al. (10) review aimed to examine the quality of existing TB-stigma intervention studies and create a novel conceptual framework of pathways to TB-stigma reduction (10). We critically appraised the methods used in this review and expanded upon the findings in the context of WHO End TB Strategy (1, 2, 11). In addition, we have examined the continuing challenges for TB-stigma interventions and how they can be refined for future implementation and scale-up.

Box 1 **TB-Stigma definitions (8).**

Enacted (or experienced) stigma encompasses the range of behaviors directly experienced by a person with TB.

Anticipated stigma is the expectation and fear of discrimination and behavior of others toward a person if they are diagnosed and/or unwell with TB, which has an impact on health-seeking behavior, whether enacted stigma occurs or not.

Internalized (or self) stigma is when those diagnosed and/or unwell with TB may accept a negative stereotype about people with TB and potentially act in a way that endorses this stereotype.

Secondary or external stigma is the negative attitude toward family members, caregivers, friends, or TB healthcare workers because they are associated with, live with, or have close contact with people with TB.

## Critical appraisal of the methods in Nuttall et al.

Nuttall et al. (10) included studies that reported the implementation and evaluation of TB-stigma reduction interventions amongst people with TB and their households, healthcare workers (HCWs) and the public. This included a wide range of study designs, with all studies importantly measuring TB-stigma. The Joanna Briggs Institute critical appraisal tool for systematic review and research synthesis (12) was used to appraise Nuttall et al. (10). The appraisal purpose was to assess methodological quality and the possibility of bias in design, conduct, and analysis of this review; following an independent process and consensus discussion two of the 11 criteria were not achieved (Table 1).

**Table 1:** Critical appraisal using the JBI critical appraisal checklist for systematic reviews and research syntheses (12).

**JBI critical appraisal checklist items**	**Responses**
Is the review question clearly and explicitly stated?	Yes, the review aimed to examine the quality, design, implementation challenges, and successes of TB-stigma intervention studies and create a novel conceptual framework of pathways to TB-stigma reduction
Were the inclusion criteria appropriate for the review question?	Yes, the review included a broad inclusion criterion that identified appropriate populations, interventions, comparators, outcomes, and study designs
Was the search strategy appropriate?	Yes, the search strategy was broad and appropriate. Appropriate keywords were used in the searches, using a controlled vocabulary of Boolean operators. The searches were focused on studies published from 1999 to 2021 and in English only
Were the sources and resources used to search for studies adequate?	Yes, databases searched included PubMed, CINAHL, Medline and Global Health. Additionally, gray literature was sourced from Google Scholar and a WHO database by “snowballing” reference tracking. However, no psychology databases were included
Were the criteria for appraising studies appropriate?	Yes, Eligible studies included those that reported the implementation and evaluation of TB-stigma reduction interventions amongst people with TB and their households, healthcare workers, and the general public, using a wide range of study designs
Was critical appraisal conducted by two or more reviewers independently?	Yes, critical appraisal was undertaken by three reviewers using a valid and reliable tool. A fourth reviewer resolved any discrepancies. The “Crowe Critical Appraisal Tool” (CCAT) (29) was used to determine the quality of the included studies. The authors developed a percentile grading of the CCAT scores based on published guidelines and existing literature
Were there methods to minimize errors in data Extraction?	No, in the article the authors outlined what data they extracted. However, the precise methods for their full paper review and data extraction remain unclear
Were the methods used to combine studies appropriate?	Yes, a narrative synthesis was undertaken although this was not clearly stated in the methods, neither was the reasoning. The review authors do state in the limitations that it was not possible to quantitatively determine the effectiveness of interventions due to methodological heterogeneity of the included studies. Qualitative data was thematically analyzed and a conceptual framework created
Was the likelihood of publication bias assessed?	No, the authors conducted a comprehensive literature search including gray literature, however, there was no formal method to minimize publication bias
Were recommendations for policy and/or practice supported by the reported data?	Yes, the recommendations for policy and/or practice state that stigma reduction activities should aim to be more inclusive of HCWs and community members, and HCWs should convey anti-stigma messages
Were the specific directives for new research appropriate?	Yes, future research should focus on reliable and valid tools are used to measure stigma, and “reduction” is considered as a key outcome and that mixed methods studies would be beneficial. They also state that the psych/social burden should be documented and advocate for a global TB stigma indicator to support research
	9/11

It is important to note that the following criteria was not met: no information that outlined any methods to minimize errors in data extraction; or evidence of a formal assessment of publication bias. The former is an important consideration to minimize bias or systematic errors in the conduct of the review. Clarity is needed to know what efforts were made by authors, for example, if data extraction was done in duplicate and independently, or evidence of training/piloting of their extraction tools (12). We can mitigate publication bias by having a comprehensive search strategy, which can best capture all relevant studies. The authors reported a detailed strategy and also looked at the gray literature, however no psychology databases were included. Publication bias was acknowledged as a potential limitation by the authors. Undertaking a meta-analysis looking at TB stigma intervention effect measurements would have been useful to test for publication bias and potential bias in outcome reporting (12). However, this may have not been possible because of the limited number of studies identified with quantitative stigma measurement tools (<10) plus the heterogeneity of the tests undertaken. Nevertheless, this influence was not discussed within the review. Overall, despite the two concerns, 1 provides a comprehensive summary of the available data that address the question of interest. However, there were no clear recommendations for their implementation.

## Results of the review by Nuttall et al.

The 11 included studies showed significant heterogeneity in design, aims, populations, type of TB-stigma targeted and regions, spanning low-income (*n* = 1), middle-income (*n* = 9), and high-income (*n* = 1) countries, respectively. Study populations varied between people with TB and their households (*n* = 5), healthcare workers (HCWs) (*n* = 3), and the public (*n* = 3). The study quality varied, with a median CCAT score of 24/40; issues such as lack of methodological detail and protocols affected lower scores. Only three studies were of high quality and review authors describe paper quality as “moderate.” Five studies measured anticipated stigma, two enacted stigma and five focused on internalized stigma. The findings showed that TB-stigma affects patients, their families, HCWs, and the public stemming from the illness, diagnosis, and treatment. There were three studies focusing on the public, three with TB HCWs and five targeting people with TB.

Interventions, like TB clubs, home visits, and psychosocial support groups significantly reduced “internalized” stigma, empowerment and changes to norms and behavior, and improved TB knowledge. However, home visits in areas with high TB-stigma, may have unintentionally triggered “anticipated” or “internalized” stigma. In addition, TB educational efforts enhanced confidence, and reduced myths.

Training for TB HCWs improved their knowledge, attitudes, and practices toward patients, contributing to better TB care. Nonetheless, TB-related HCWs frequently faced stigma from their peers. While the training did not reduce “secondary or external” stigma, TB-related HCWs could potentially utilize campaign materials to educate their local communities. However, in public health interventions, the failure to deliver a clear health message, through educational material, allowed TB-stigma misconceptions to persist or worsen.

Some studies employed quantitative questionnaires to measure TB-stigma, with the number of questions varying from 3 to 14. Half of these studies used validated TB-stigma tools. The others used adapted tools, and one piloted a new tool in six different African communities. Some studies utilized qualitative methods, such as focus groups, interviews, and observations, to assess stigma. These focused on exploring how TB patients coped with “internalized” stigma, “anticipated” stigma from others, and one study focused on, “secondary or external,” how HCWs working with TB patients experienced stigmatization from their colleagues. Challenges related to implementation, delivery, and process indicators (e.g., fidelity, acceptability, and feasibility) were rarely addressed in the studies.

By synthesizing the interventions and outcomes of these studies, Nutall et al. (10), designed a conceptual framework with pathways to reduce TB-stigma based on targeted populations (10). In this, they highlighted mechanisms for intended outcomes, which included improving understanding, removing misconceptions, attitude and behaviors change among public via educational material, mass gathering (health talks) and health education programs. They also highlighted intended impacts in terms of reducing stigma among people with TB, toward TB-HCWs, TB “internalized” stigma, improving TB treatment adherence, completion, success and minimizing the economic consequences of TB. However, the authors did not make recommendations on how to use this framework.

## Discussion

In global efforts and strategies to prevent and control tuberculosis, stigma has historically been a low-priority issue (13). At its inception the WHO End TB strategy did not explicitly talk about stigma but emphasized addressing social determinants of TB via stakeholder involvement at individual, community, and government levels through patient-centered care approaches, and health education and awareness (2). Combating stigma and discrimination was included in the updated “Implementing the End of TB Strategy” (11). TB-Stigma was the sixth priority recommendation but still failed to emphasize how to introduce mechanisms to report, address and evaluate the stigma status among tuberculosis patients at a system level.

TB stigma not being a primary objective of policy has led to gaps in the literature. Systematic reviews in this field predominantly feature qualitative evidence (9, 10, 14, 15) consisting of studies majorly from Africa, although most HBC for TB, including Pakistan, are found in Asia (1). Nuttall et al. (10) reviewed the literature and created a conceptual framework that provides a useful guide to understanding the pathways needed in TB-stigma reduction interventions. Decreasing TB stigma requires a multi-level, holistic approach, with community-informed, person-centered interventions prioritized by TB programs (16). However, Nuttall et al. make no explicit mention of how TB stigma-reduction efforts should be integrated within existing health systems.

### Empowering people with TB, and communities

Nuttall et al. (10) emphasized intellectual and emotional empowerment. In high-risk populations, the framework effectively highlights the need for educational, emotional, and psychosocial support for TB patients, HCWs, and the community. TB-stigma interventions seem to lean heavily on informational interventions such as pamphlets, health talks and workshops. These methods alone may not fully address deep-rooted stigma, particularly where poverty and cultural beliefs or systemic issues are involved (17, 18). For example, though the framework includes public education, it doesn't explicitly mention adapting interventions to local cultural contexts, particularly in LMICs (19). Few interventions have engaged patients and communities effectively at each step of the care process, highlighting the need for further research on prioritizing patient-centered care in resource-constrained settings (20).

### Addressing the social determinants of TB

The framework touches on misconceptions and psychosocial factors but does not fully address structural barriers like poverty, healthcare access, and discrimination. Addressing TB-stigma requires tackling these broader determinants of health, especially in resource-constrained settings (19). While Nuttall et al. (10) addresses capability through knowledge and emotional support, it overlooked the critical aspect of financial empowerment. Providing financial empowerment is essential to ensure opportunity, which enables patients to access and utilize TB care services. For example, asking patients to wear masks or attend clinics is ineffective if they lack the financial means to buy masks or cover transportation costs. Without financial support, even well-informed patients may struggle to engage in care. Therefore, offering both knowledge and the means to act on that knowledge is equally important for successful TB stigma reduction and treatment adherence.

### Health system integration and strengthening

There is a need for greater emphasis on strengthening health systems in HBC for TB, which currently lag due to limited focus and resources. Anticipated, internal, and enacted stigma are significant barriers to TB care, directly affecting patient wellbeing, yet targeted stigma interventions remain limited (5). Understanding stigma drivers is essential to enhance timely diagnosis and treatment for people living with TB, with stigma reduction being critical to advancing care engagement (21). To this end measuring all forms of stigma is important. System-level enhancements such as routine stigma monitoring mechanisms, integration of stigma indicators into patient records, and structured monitoring and evaluation processes contribute to sustained health system strengthening. However, validated tools to measure TB-stigma remain scarce (22) and those used lack cultural and linguistic validation, highlighting the critical need for reliable and valid tools to measure TB stigma in LMICs most affected by the disease (7).

In addition, stigma from healthcare workers who don't work in TB facilities not only impacts patient care but also health-seeking behavior, necessitating workforce stigma-reduction strategies to improve patient outcomes (23). Nuttall et al. (10) highlighted the stigma faced by HCWs, highlighting the need to make HCWs feel safe while undergoing clinical training and psychosocial support. To be effective, stigma reduction interventions must be part of a larger health system response, including mental health services, social support systems and public health programs. Furthermore, workplace protections and gender-sensitive support are added to ensure equitable, non-discriminatory environments that acknowledge social and gender-based vulnerabilities (24, 25). However, confidentiality safeguards and mental health support for both healthcare workers and people with TB should be included to foster trust, psychological wellbeing, and a stigma-free care environment (26).

### Navigating emerging, operationalising, and future interventions

The recent WHO report highlights progress in European and African countries, (1) demonstrating that targeted efforts can yield results. However, there is a need for greater emphasis on strengthening systems and research activities in other HBC for TB, like Pakistan, which currently lag due to limited focus and resources. In addition, research should aim to characterize TB stigma across diverse populations, using validated tools to assess its impact on time to diagnosis, treatment adherence, morbidity, and mortality, while also developing new strategies to mitigate TB stigma.

Current interventions focus on raising community awareness, providing patient counseling on problem-solving and emotional skills, creating culturally sensitive and scientifically accurate media messages, incentives, and enhancing healthcare professionals' empathy, respect, concern, and cultural sensitivity (13). TB stigma in communities can be reduced through shared commitment to TB prevention and supportive environments at home, in the community, and within healthcare services (23).

With most of the studies, in Nuttall et al. (10), being reported from Africa and earlier than 2021, it is highly recommended to search for recent and contextual interventions to take insights for developing innovative and context-based solutions. For example, a communication intervention based on self-efficacy and social support theories at Thai high schools (27) and “Jaga rasa Jaga tangga” (take care of your neighbors as well as take care of their feelings) community for HCWs (23). The diverse contexts, health infrastructure, and HBC for TB require tailored approaches to effectively address TB prevention and control efforts in different settings (19).

### Limitations

We followed the steps outlined in the Rapid Conversion of Evidence Summaries (RaCES) developed by the NIHR Applied Research Collaboration North West Coast (28). Through this approach we aim to answer an important health and social care question to help inform policy and practice and aid implementation. Therefore, we acknowledge that our perspective does not present findings from an original systematic review or a formal literature update, but is a critical reflection based primarily on existing evidence in a recently published systematic review. This approach is focused on implementing the best current knowledge in the real world, supported and substantiated through relevant and credible references to ensure conceptual validity and evidence-based interpretation.

## Conclusions

In this perspective, we examined the continuing challenges and outlined potential changes to this framework making it a better guide for future implementation and scale-up in HBC for TB. To move closer to eradicating TB, we require urgent, well-organized, and committed multi-sectoral actions to reducing TB stigma, that extend beyond national and global TB programs. This effort must be supported by substantial investments in research and the equitable, rapid implementation of innovations worldwide. Few TB stigma reduction interventions have been rigorously evaluated, and replication of effective studies has been rare. The conceptual framework by Nuttall et al. (10) is a useful guide helping HBC for TB to operationalise effective TB-stigma reduction programs.

## Data Availability

The original contributions presented in the study are included in the article/supplementary material, further inquiries can be directed to the corresponding author.
